# Predictive value of preoperative ultrasonographic measurement of gastric morphology for the occurrence of postoperative nausea and vomiting among patients undergoing gynecological laparoscopic surgery

**DOI:** 10.3389/fonc.2024.1296445

**Published:** 2024-07-23

**Authors:** Weiji Qiu, Jun Yin, Huazheng Liang, Qiqing Shi, Chang Liu, Lina Zhang, Gang Bai, Guozhong Chen, Lize Xiong

**Affiliations:** ^1^ Department of Anesthesiology and Perioperative Medicine, Shanghai Key Laboratory of Anesthesiology and Brain Functional Modulation, Clinical Research Center for Anesthesiology and Perioperative Medicine, Translational Research Institute of Brain and Brain-Like Intelligence, Shanghai Fourth People’s Hospital, School of Medicine, Tongji University, Shanghai, China; ^2^ Department of Anesthesiology, Parkway Shanghai Hospital, Shanghai, China; ^3^ Department of Ultrasound Medicine, Shanghai Fourth People’s Hospital, School of Medicine, Tongji University, Shanghai, China; ^4^ Monash Suzhou Research Institute, Suzhou, Jiangsu, China; ^5^ Department of Medical Ultrasound, Shanghai Tenth People’s Hospital, Shanghai, China; ^6^ Department of Biostatistics, Clinical Research Institute, Shanghai Jiao Tong University School of Medicine, Shanghai, China

**Keywords:** gastrointestinal tract, preoperative morphological analysis, muscularis propria, postoperative nausea and vomiting, ultrasonography

## Abstract

**Background:**

Pre-operative prediction of postoperative nausea and vomiting (PONV) is primarily based on the patient’s medical history. The predictive value of gastric morphological parameters observed on ultrasonography has not been comprehensively assessed.

**Methods:**

A prospective observational study was conducted to evaluate the pre-operative ultrasonographic measurement of gastric morphology for predicting PONV. The gastric antrum of the participants was assessed using ultrasound before anesthesia, and the occurrence of PONV in the first 6 hours and during the 6–24 hours after surgery was reported. The main indicators included the thickness of the muscularis propria (TMP) and the cross-sectional area of the inner side of the muscularis propria (CSA-ISMP). These were recorded and analyzed. Logistic regression analysis was applied to identify factors for PONV.

**Results:**

A total of 72 patients scheduled for elective gynecological laparoscopic surgery were investigated in the study. The pre-operative CSA-ISMP of patients with PONV in the first 6 hours was significantly greater than that of those without PONV (2.765 ± 0.865 cm² vs 2.349 ± 0.881 cm², P=0.0308), with an area under the curve of 0.648 (95% CI, 0.518 to 0.778, P=0.031). Conversely, the pre-operative TMP of patients with PONV during the 6–24 hours was significantly smaller than that of those without PONV (1.530 ± 0.473 mm vs 2.038 ± 0.707 mm, P=0.0021), with an area under the curve of 0.722 (95% CI, 0.602 to 0.842, P=0.003). Logistic regression analysis confirmed that CSA-ISMP was an independent risk factor for PONV in the first 6 hours (OR=2.986, P=0.038), and TMP was an independent protective factor for PONV during the 6–24 hours after surgery (OR=0.115, P=0.006).

**Conclusion:**

Patients with a larger pre-operative CSA-ISMP or a thinner TMP are prone to develop PONV in the first 6 hours or during the 6–24 hours after surgery, respectively.

**China clinical trial registration center:**

http://www.chictr.org.cn (ChiCTR2100055068).

## 1 Introduction

Postoperative nausea and vomiting (PONV) is the second most common postoperative complication, often referring to nausea and/or vomiting, or retching within 24 hours after surgery. It imposes billions of dollars in costs on the healthcare system annually (1–3). It is widely accepted that surgical factors (type and duration of surgery) (4, 5), anesthesia factors (inhaled anesthetics, nitrous oxide, postoperative opioid consumption) (6–8), and patient demographics (age, gender, smoking history, history of PONV or motion sickness) (9, 10) influence the incidence of PONV. The Apfel score, commonly used in clinical practice to evaluate the risk of developing PONV in surgical patients, considers gender, smoking history, history of PONV or motion sickness, and postoperative opioid consumption (9, 11). However, it remains unclear why some patients without risk factors develop PONV while others with multiple risk factors do not, despite receiving the same anesthesia and surgical interventions (9). If PONV is not solely related to the unmodifiable characteristics of the medical condition and the surgical anesthesia process, it is crucial to explore whether other factors might influence the occurrence of PONV post-surgery.

Ultrasound is a commonly used method to examine solid organs in the abdomen. Recently, it has been employed to detect the shape and function of the gastrointestinal tract, though it was once considered an insurmountable challenge for ultrasonography due to its irregular shape, residual contents, and gas accumulation, which hinder imaging (12–14). With advancements in understanding gastrointestinal morphology and improvements in ultrasonography and imaging resolution in recent years, gastrointestinal ultrasonography can now be used to assess gastrointestinal tumors during follow-up (15, 16), evaluate gastric emptying function (17, 18), assess gastric peristalsis (19, 20), and determine the nature (liquid, solid, or mixed) and volume of gastric contents in perioperative patients (17, 21). However, few studies have investigated the value of preoperative gastric morphology in predicting the occurrence of PONV.

It is known that excessive content in the gastrointestinal tract can cause nausea and vomiting. Similarly, disturbances in the functional state of the gastrointestinal tract, such as abnormal peristalsis frequency, also lead to nausea and vomiting (22, 23). Functional changes in the tissue are often accompanied by structural changes (24). Gastric emptying primarily depends on the coordinated movements of the muscularis propria, with muscle contractile strength often correlated with muscle thickness. Studies have shown that estimating gastric content using the cross-sectional area of the gastric antrum can predict the occurrence of postoperative vomiting (25). Given the accuracy of identifying the muscularis propria via ultrasound, this study measured the thickness and cross-sectional area of the muscularis propria in the gastric antrum to assess the risk of PONV. In this study, we hypothesized that differences in preoperative gastrointestinal morphological parameters could predict PONV.

We conducted a prospective observational study, utilizing ultrasound to assess the gastrointestinal morphological parameters of patients undergoing gynecological laparoscopic surgery before anesthesia. We then compared these parameters between patients who experienced PONV and those who did not. Finally, we evaluated the predictive value of these parameters for PONV. This study provides a novel foundation for more accurate clinical prevention and treatment of PONV.

## 2 Materials and methods

### 2.1 Ethics approval and consent to participate

This prospective observational study received approval from the Ethics Committee of Shanghai Fourth People’s Hospital Affiliated to Tongji University (No. 2020076–001) in Shanghai, China. It was retrospectively registered on the website of the China Clinical Trial Registration Center (registration number ChiCTR2100055068). Patients were informed about the procedures, and written informed consent was obtained from each participant the day before their operation. This research adhered strictly to the ethical standards outlined in the Helsinki Declaration. To protect patient privacy, the demographic information of all participants was anonymized during analysis.

### 2.2 Patient identification and selection

Patients scheduled for elective laparoscopic ovarian and/or uterine surgery between April 2021 and February 2022 in the Gynecology Department of Shanghai Fourth People’s Hospital, School of Medicine, Tongji University, were enrolled in the present study. The inclusion criteria were: 1) age between 18 and 70 years, 2) ASA physical status I or II, 3) BMI between 18.5 and 35, and 4) ability to understand the study protocol and voluntary signing of the informed consent form. The exclusion criteria were: 1) upper gastrointestinal tract anatomical abnormalities (such as a history of upper gastrointestinal surgery), 2) history of gastrointestinal inflammation within the past 6 months, 3) use of medications affecting gastric motility within the past month, 4) delayed gastric emptying, 5) pregnancy, 6) poorly controlled diabetes, 7) difficulties in determining the gastric antrum during the procedure, and 8) inability to comply with the study protocol for other reasons.

### 2.3 Study procedure

#### 2.3.1 Preparation of anesthesia

Demographic information and concurrent medical conditions, including age, gender, weight, height, BMI, history of diseases, current diagnosis and treatment, smoking, and history of PONV or motion sickness, were collected during the preoperative visit. Patients were instructed to fast for at least 6 hours before the operation, with an allowance of up to 200 ml of non-carbonated clear liquids up to 2 hours before the procedure. Prior to anesthesia, the shape and size of the gastric antrum, as well as the thickness of the muscularis propria, were measured using ultrasound while patients were in the supine position. Ultrasound images were dynamically recorded for 6 minutes after clearly differentiating the gastric antrum from surrounding structures.

#### 2.3.2 Anesthesia management

Patients were induced into an anesthetic state using a sequential induction method with sufentanyl (0.3–0.5 μg/kg), propofol (2 mg/kg), and rocuronium bromide (0.6 mg/kg). Assisted positive ventilation was maintained at a pressure not exceeding 20 cmH_2_O to minimize gastric distension during anesthesia induction. Once sedation and muscle relaxation were sufficient, tracheal intubation was performed, and patients were ventilated with a tidal volume of 6 ml/kg in volume control mode and a fresh gas flow rate of 3 L/min. Anesthesia was maintained with a combination of intravenous and inhalational agents. Sevoflurane was inhaled continuously to achieve a minimum alveolar concentration (MAC) of 0.8, sufentanyl (0.1–0.2 μg/kg) was administered every half hour, and additional rocuronium (0.15 mg/kg) was added to maintain a train-of-four (TOF) ratio of less than 10%, with muscle relaxation monitored every 20 seconds to ensure heart rate and blood pressure fluctuations remained within 20% of baseline values. Tidal volume and respiratory rate were adjusted to keep the end-tidal carbon dioxide partial pressure between 35 and 45 mmHg and airway pressure below 30 cmH_2_O. After surgery, patients were not awakened until the MAC dropped below 0.2 and the TOF ratio increased to 90% or above. Consciousness and muscle relaxation recovery were checked again before tracheal extubation, and patients were transferred to the post-anesthesia care unit (PACU) for an additional one-hour observation before returning to the ward. Postoperative analgesia was managed with parecoxib and bilateral transversalis fascia nerve block under ultrasound guidance using 20 ml of 0.25% ropivacaine on each side.

#### 2.3.3 Postoperative follow-up

The general conditions of the patients, first postoperative exhaust time, and the occurrence of PONV were closely monitored based on a modified protocol from previous studies (6, 26) and recorded at the 6th hour (T1) and 24th hour (T2) after surgery. Patients were classified as having PONV if they experienced postoperative vomiting or reported nausea with a score greater than zero on an 11-point scale during the study period. PONV was subsequently used as a binary classification variable (yes/no) for logistic regression analysis. If a patient developed PONV, 10 mg of metoclopramide was prescribed. If symptoms persisted, an additional dose was administered 6 hours later.

### 2.4 Gastric ultrasonography and analysis

#### 2.4.1 Gastric ultrasonography

**Figure 1:**
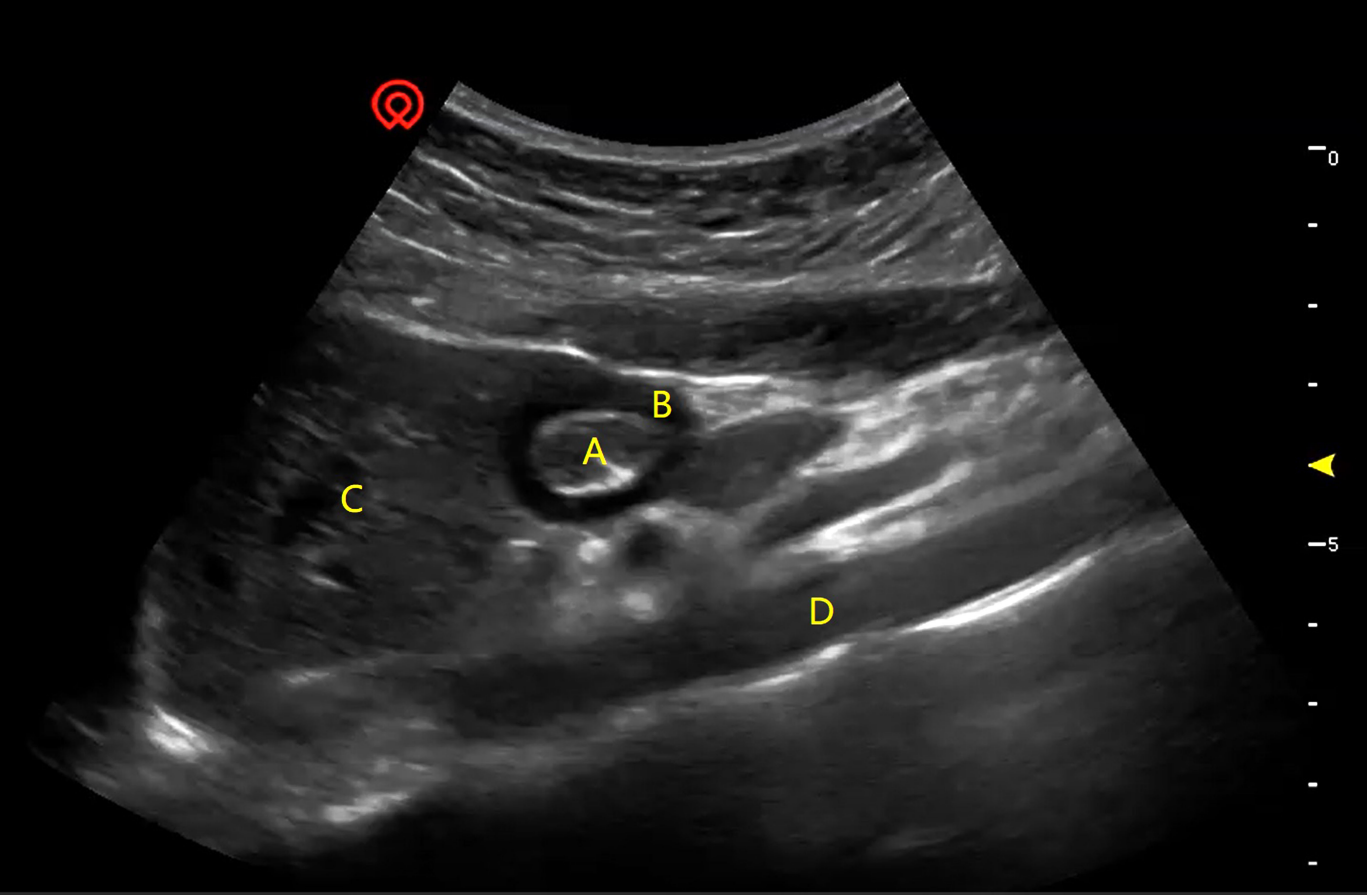
A typical image of preoperative ultrasonography of the gastric antrum and surrounding structures. **(A)** CSA-ISMP, the cross-sectional area of the inner side of the muscularis propria; **(B)** TMP, the thickness of the muscularis propria; **(C)** the left hepatic lobe; **(D)** the aorta.

An anesthesiologist, experienced in independently identifying the gastric antrum and surrounding structures in at least 50 patients, performed the ultrasonographic examination. This was conducted under the supervision and guidance of two professional ultrasonographers while the patient was in the supine position before anesthesia induction. A Navi s Ultrasound Machine (specialized ultrasound in anesthesiology) produced by Shenzhen Wisonic Medical Technology Co. Ltd (China) and a C5–1B low-frequency convex array probe (1 to 5 MHz) were used for examination. The gastric antrum was localized in the subxiphoid region on sagittal or parasagittal scanning, with the left lobe of the liver, the abdominal aorta, and the superior mesenteric artery as key landmarks (17, 27). Once the gastric antrum was confirmed, scanning commenced, and images from the 6-minute scan were saved for independent postoperative analysis by two physicians (**Figure 1**).

#### 2.4.2 Main indicators

The main parameters included the thickness of the muscularis propria (TMP) and the cross-sectional area of the inner side of the muscularis propria (CSA-ISMP) of the gastric antrum in the supine position before the induction of anesthesia. Both TMP and CSA-ISMP of the gastric antrum were measured when the gastric antrum was relatively stationary at two different time points. The anteroposterior and craniocaudal diameters, measured perpendicular to each other on the inner side of the muscularis propria of the gastric antrum, were used to calculate the CSA-ISMP using the formula described by Bolondi (28):


$$\begin{array}{c} \mathrm{CSA}-\mathrm{ISMP}\;(\mathrm{cross}\\ -\mathrm{sectional}\;\mathrm{area}\;\mathrm{of}\;\mathrm{the}\;\mathrm{inner}\;\mathrm{side}\;\mathrm{of}\;\mathrm{muscularis}\;\mathrm{propria})\;\left(cm^{2}\right)\\ =\;\mathrm{Anteroposterior}\;\mathrm{Diameter}\;\left(\mathrm{cm}\right)\\ \times \;\mathrm{Craniocaudal}\;\mathrm{Diameter}\;\left(\mathrm{cm}\right)\times \pi /4 \end{array}$$

#### 2.4.3 Secondary indicators

Secondary parameters included the frequency of peristalsis (FP) during a 6-minute period of ultrasound imaging, residual gastric content, and gas accumulation. FP was determined as the number of peristaltic waves counted by two physicians upon reviewing the 6-minute ultrasound recording. Residual gastric content and gas accumulation were independently assessed by these two physicians. In cases where their assessments differed, a third physician was consulted to make the final decision.

### 2.5 Statistical analysis

Due to the lack of previous studies on predicting PONV using preoperative ultrasonographic assessments of the gastric antrum, we determined our sample size based on key indicators: TMP and CSA-ISMP, along with established risk factors for PONV such as age, gender, type of surgery, history of PONV or motion sickness, smoking history, operation duration, intraoperative inhalational anesthetics, and postoperative opioid use (2). Gender, type of surgery, and intraoperative inhalational anesthetics are inherent variables. Our clinical experience with gynecological laparoscopic surgery suggests that a combination of local nerve block and oral NSAIDs effectively manages postoperative pain without necessitating additional opioids. Therefore, opioids were not administered postoperatively in our study. Based on prior research and institutional data, we estimated the overall incidence of PONV in gynecological laparoscopic procedures to be around 50% (4). Allowing for a 20% dropout rate, we aimed to recruit a minimum of 75 patients for this study.

Continuous variables were presented as mean ± standard deviation (95% CI) or median (25th to 75th quartiles), depending on their distribution. Normally distributed data were analyzed using Student’s t-test, while non-normally distributed data were assessed with the Mann-Whitney U test. Categorical variables were expressed as frequencies and percentages, and differences were evaluated using the Chi-square test, with odds ratios (OR) and 95% confidence intervals (CI) calculated accordingly. Receiver Operating Characteristic (ROC) curves were constructed, and the area under the curve (AUC) was determined to assess the predictive capability of TMP and CSA-ISMP for PONV occurring within the first 6 hours or between 6 to 24 hours post-surgery. Logistic regression analysis was performed to estimate relative risks based on established PONV factors. Statistical significance was defined as P < 0.05. Data analyses were conducted using SPSS (version 19), GraphPad Prism 8.0, and Microsoft Excel 365. The reporting format adhered to the STROBE guidelines (29).

## 3 Results

### 3.1 Patient characteristics

**Figure 2:**
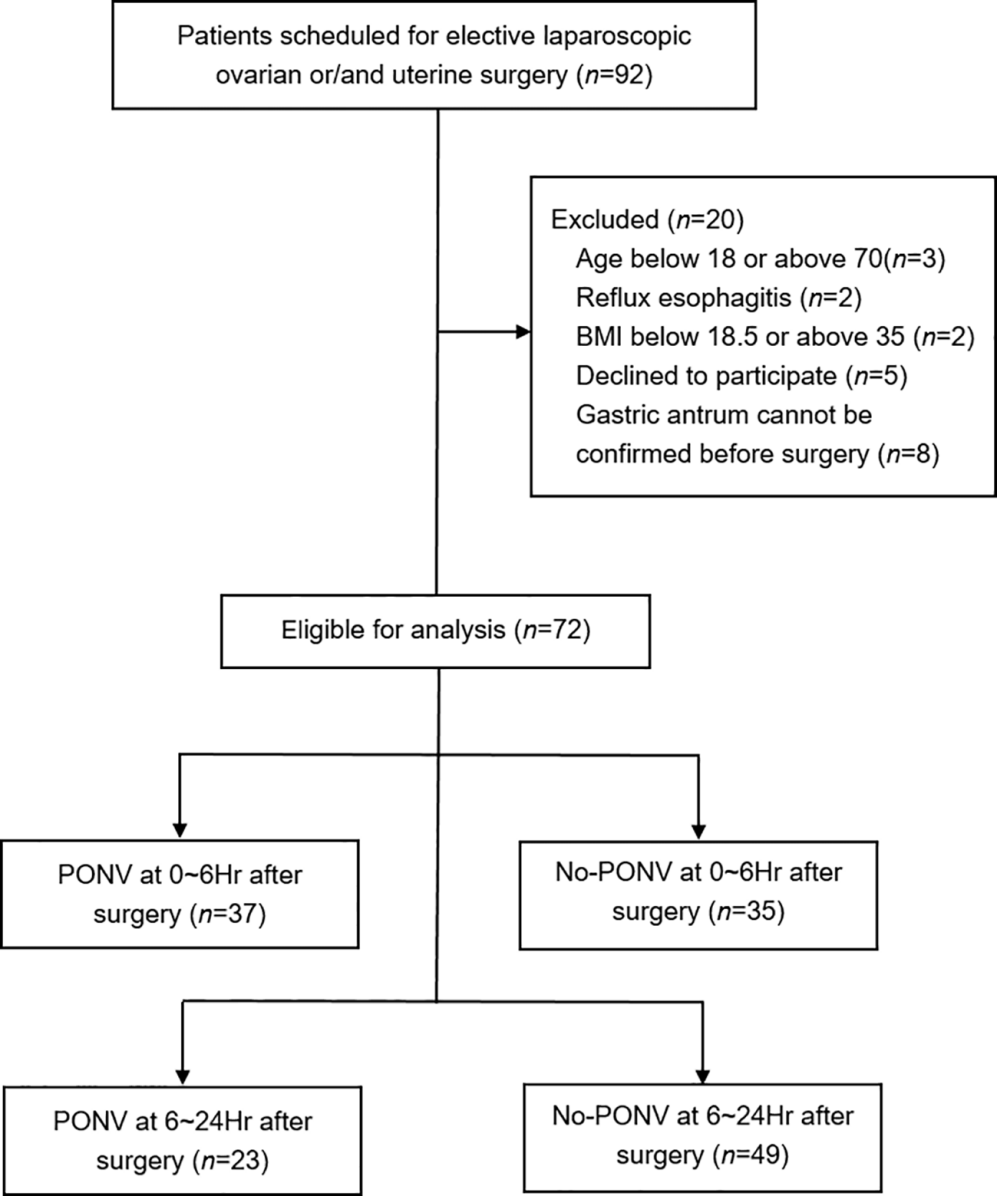
Flowchart depicting patient recruitment and analysis.

A total of 92 patients initially participated in the assessment. Twenty patients were excluded from the study: seven due to exceeding the specified age and weight limits or having existing medical conditions, five who declined to provide informed consent, and eight whose gastric antrum could not be identified during examination or analysis. Among the 72 patients included in the final analysis, 37 (51.39%) experienced PONV within the first 6 hours post-operation, and 23 (31.94%) between 6 to 24 hours after their operation (**Figure 2**).

There were no significant differences observed in age, height, weight, BMI index, smoking history, history of PONV or motion sickness (MS), or postoperative opioid consumption between those who developed PONV and those who did not during either the 0 to 6-hour or 6 to 24-hour periods after surgery (**Table 1**).

**Table 1:** Comparison of patients’ preoperative characteristics.

	0~6Hr PONV			6~24Hr PONV		
Variables	Yes (*n*=37)	No (*n*=35)	*P*	Yes (*n*=23)	No (*n*=49)	*P*
Age (years)	43.95 ± 9.94	44.34 ± 11.44	0.8754	44.04 ± 10.67	44.18 ± 10.71	0.9588
Height (cm)	162.3 ± 5.2	160.2 ± 5.7	0.1055	161.3 ± 5.3	161.3 ± 5.6	0.9778
Weight (kg)	62.65 ± 9.16	60.21 ± 10.32	0.2928	61.37 ± 8.64	61.51 ± 10.31	0.9550
BMI (kg/m^2)^	23.74 ± 2.89	23.38 ± 3.16	0.6170	23.55 ± 2.84	23.57 ± 3.11	0.9762
History of						
Smoking (%)	3 (8.1)	1 (2.9)	0.6148	0 (0)	4 (8.2)	0.2989
PONV or MS (%)	6 (16.2)	5 (14.3)	0.8200	5 (21.7)	6 (12.2)	0.2965

Continuous variables are summarized as mean ± standard deviation; Categorical data are summarized as n (column %).

PONV, postoperative nausea and vomiting; BMI, body mass index; MS, motion sickness.

### 3.2 TMP of gastric antrum in resting state before operation predicts PONV at 6~24 hours after operation

**Figure 3:**
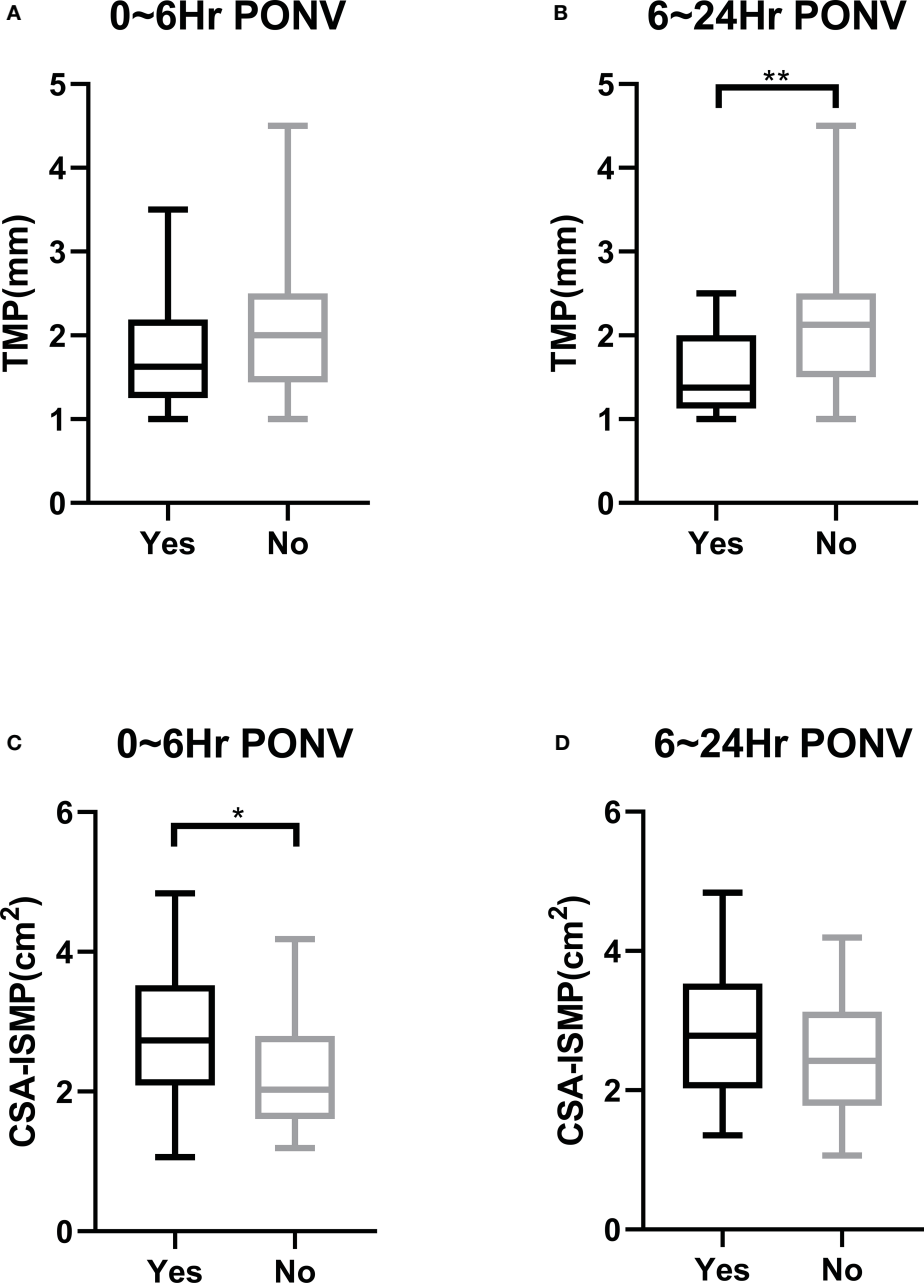
Comparison of morphological parameters of the gastric antrum on ultrasound in the resting state before the induction of anesthesia between patients with and without PONV. **(A)** Preoperative TMP in patients with or without PONV in the first 6 hours after operation. **(B)** Preoperative TMP in patients with or without PONV during the period of 6~24 hours after operation. **(C)** Preoperative CSA-ISMP in patients with or without PONV in the first 6 hours after operation. **(D)** Preoperative CSA-ISMP in patients with or without PONV during the period of 6~24 hours after operation. * indicates P<0.05; ** indicates P<0.01.

**Figure 4:**
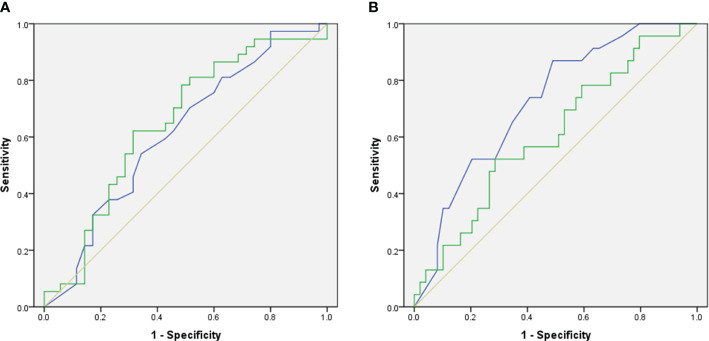
The predictive value of preoperative ultrasonographic morphological parameters of the gastric antrum before the induction of anesthesia in predicting the occurrence of PONV. **(A)** In the first 6 hours after operation. **(B)** During the period of 6~24 hours after operation. The blue line indicates the predicted probability of TMP, the green line indicates the predicted probability of CSA-ISMP, and the orange line is a reference line.

There was no statistically significant difference in the TMP of the gastric antrum before anesthesia between patients who did and did not develop PONV within the first 6 hours post-operation (1.740 ± 0.595 mm vs 2.020 ± 0.744 mm, P=0.0978) (**Figure 3A**). However, patients who experienced PONV during the 6~24 hours after surgery had a significantly thinner TMP compared to those who did not (1.530 ± 0.473 mm vs 2.038 ± 0.707 mm, P=0.0021) (**Figure 3B**). ROC curve analysis indicated an area under the curve of 0.614 (95% CI, 0.482 to 0.745, P=0.098) for predicting PONV within the first 6 hours after surgery (**Figure 4A**), and an area under the curve of 0.722 (95% CI, 0.602 to 0.842, P=0.003) for predicting PONV during the 6~24 hours post-operation (**Figure 4B**). These results suggest that TMP of the gastric antrum before anesthesia induction can predict the occurrence of PONV during the 6~24 hours post-surgery period. According to the Youden’s index of the ROC curve, the optimal preoperative TMP for predicting PONV during the 6~24 hours post-surgery was 2.063 mm, with a relative risk of 6.944 (OR=6.9, 95% CI: 1.977 to 23.82, P=0.002). The sensitivity, specificity, positive predictive value, and negative predictive value were 86.96%, 51.02%, 45.45%, and 89.29%, respectively. Interobserver variability and agreement were analyzed (**Supplementary Table 1**).

### 3.3 CSA-ISMP of gastric antrum predicts PONV at 0~6 hours after operation

The CSA-ISMP measured before anesthesia induction in patients who developed PONV within the first 6 hours after surgery was significantly larger compared to those who did not (2.765 ± 0.865 cm² vs 2.349 ± 0.881 cm², P=0.0308) (**Figure 3C**). However, there was no statistical difference in CSA-ISMP between patients who did and did not develop PONV during the 6~24 hours post-operation period (2.795 ± 0.922 cm² vs 2.454 ± 0.864 cm², P=0.1305) (**Figure 3D**). The ROC curve analysis indicated an area under the curve of 0.648 (95% CI, 0.518 to 0.778, P=0.031) for predicting PONV occurrence within the first 6 hours after surgery (**Figure 4A**), and an area under the curve of 0.608 (95% CI, 0.469 to 0.747, P=0.142) for predicting PONV during the 6~24 hours post-operation (**Figure 4B**). According to the Youden’s index of the ROC curve, the optimal predictive value for CSA-ISMP in predicting PONV within the first 6 hours after surgery was 2.579 cm², with a relative risk of 3.584 (OR=3.58, 95% CI: 1.402 to 8.985, P=0.009). The sensitivity, specificity, positive predictive value, and negative predictive value were 62.16%, 68.57%, 67.65%, and 63.16%, respectively. Interobserver variability and agreement were analyzed (**Supplementary Table 1**).

### 3.4 Patients with abnormal gastric peristalsis are prone to develop PONV at 6~24 hours after operation

Using preoperative ultrasonography to assess gastric peristalsis in the gastric antrum over a 6-minute period, the median frequency (25th to 75th quartiles), determined by two physicians, was 2 (0 to 4). Based on this, patients were categorized into two groups: those with a frequency of peristalsis (FP) between 1 and 4 were grouped as F_1~4_, while those with an FP of 0 or greater than 4 were grouped as F_0or>4_. It was observed that the relative risk of PONV between the F_0or>4_ and F_1~4_ groups within the first 6 hours after surgery was 1.552 (OR=1.55, 95% CI: 0.627 to 3.995, P=0.3579). However, during the 6~24 hours post-operation period, the relative risk increased to 2.951 (OR=2.95, 95% CI: 0.977 to 9.058, P=0.0463). The sensitivity, specificity, positive predictive value, and negative predictive value were 73.91%, 51.02%, 41.46%, and 80.65%, respectively. These findings indicate that patients with either excessively slow or fast preoperative antral FP are at higher risk of developing PONV during the 6~24 hours after operation (**Table 2**).

**Table 2:** Associations between preoperative ultrasonographic detection of antral peristalsis frequency, gastric residue, and gas accumulation, and the occurrence of PONV within the 0–6 hour and 6–24 hour periods after surgery.

	PONV				
0~6Hr	Yes (*n*=37)	No (*n*=35)	OR	95% CI	*P*
FP_0or>4_ (%)	23(62.2)	18(51.4)	1.552	0.627, 3.995	0.3579
GR (%)	23(62.2)	16(45.7)	1.951	0.794, 5.094	0.1615
GA (%)	19(51.4)	14(40.0)	1.583	0.647, 4.051	0.3340
6~24Hr	Yes (*n*=23)	No (*n*=49)	OR	95% CI	*P*
FP_0or>4_ (%)	17(73.9)	24(49.0)	2.951	0.977, 9.058	0.0463
GR (%)	15(65.2)	24(49.0)	1.953	0.690, 5.748	0.1973
GA (%)	12(52.2)	21(42.9)	1.455	0.512, 3.662	0.4594

PONV, postoperative nausea and vomiting; FP_0or>4_, the calculated frequency of peristalsis was 0 or more than 4 times; GR, gastric residuals; GA, gastric air accumulation.

Categorical data are summarized as *n* (column %).

### 3.5 Residual content or gas accumulation in the gastric antrum does not predict the occurrence of PONV

The presence of gastric residue before anesthesia was assessed independently by two physicians, with a consensus reached in 48 patients (66.67%). A third physician resolved any discrepancies before statistical analysis. It was determined that there was no significant difference in the incidence of gastric residue between patients who developed PONV and those who did not, either within the first 6 hours (23 vs 16, OR=1.95, 95% CI: 0.794 to 5.094, P=0.1615) or during the 6~24 hours period (15 vs 24, OR=1.95, 95% CI: 0.690 to 5.748, P=0.1973) post-operation (**Table 2**).

Similarly, gas accumulation in the gastric antrum before anesthesia induction was assessed by two physicians, with agreement in 59 patients (81.94%). No significant difference was observed in the prevalence of gas accumulation between patients who developed PONV and those who did not, either within the first 6 hours (19 vs 14, OR=1.58, 95% CI: 0.647 to 4.051, P=0.3340) or during the 6~24 hours period (12 vs 21, OR=1.45, 95% CI: 0.512 to 3.662, P=0.4594) post-operation (**Table 2**).

### 3.6 Predictive value of preoperative morphological factors for PONV

Logistic regression analysis was conducted on factors known to influence the incidence of PONV within the first 6 hours or during the 6~24 hours post-operation period: age, operation duration, smoking history (smoker=1, nonsmoker=0), history of PONV or motion sickness (yes=1, no=0), TMP (thick=1, thin=0), CSA-ISMP (large=1, small=0), and FP (F_0or>4_ = 1, F_1~4 _= 0) (**Table 3**). The analysis revealed that TMP was an independent protective factor against PONV during the 6~24 hours post-surgery, with an estimated relative risk of 0.115 (OR=0.115, 95% CI: 0.024 to 0.544, P=0.006). Conversely, CSA-ISMP emerged as an independent risk factor for PONV occurring within the first 6 hours after operation, with an estimated relative risk of 2.986 (OR=2.99, 95% CI: 1.061 to 8.404, P=0.038).

**Table 3:** Multivariate logistic regression analysis revealed the independent predictors of PONV.

	0~6Hr			6~24Hr		
Variables	OR	95% CI	*P*	OR	95% CI	*P*
Surgery period	1.010	1.000, 1.021	0.058	1.017	1.004, 1.030	0.010
Age	0.990	0.941, 1.041	0.693	0.975	0.914, 1.041	0.451
Smoking history	3.325	0.280, 39.53	0.341	0.000	0.000, -	0.999
PONV history	0.894	0.217, 3.679	0.876	1.393	0.304, 6.387	0.670
CSA-ISMP	2.986	1.061, 8.404	0.038	0.929	0.258, 3.347	0.911
TMP	0.564	0.195, 1.630	0.290	0.115	0.024, 0.544	0.006
FP_0or>4_	1.536	0.537, 4.397	0.424	3.510	0.941, 13.10	0.062

PONV, postoperative nausea and vomiting; CSA-ISMP, the cross-sectional area of the inner side of muscularis propria; TMP, the thickness of the muscularis propria.

FP_0or>4_, the calculated frequency of peristalsis was 0 or more than 4 times.

## 4 Discussion

The gastric antrum consists histologically of the serosa, muscularis propria, submucosa, muscularis mucosae, and mucosal layers. Kimmey et al. first described this five-layer structure of the gastric wall using ultrasound in 1989 (30). However, due to the thinness of the muscularis mucosa and the interaction of the submucosa, muscularis mucosa, and mucosal layers with residual gas or contents in the fasting state, accurate identification of these layers using ultrasound is challenging (15). Moreover, the use of a low-frequency probe is often necessary to distinguish the gastric antrum from surrounding tissues during ultrasonography, further complicating the identification of these layers (25).

In contrast, the muscularis propria of the gastric antrum, comprising three layers of smooth muscles—inner oblique, middle circular, and outer longitudinal—exhibits high elasticity. The coordinated contraction of these muscle layers generates rhythmic peristalsis in the gastrointestinal tract, facilitating food grinding (31, 32). In the fasting state, these muscle layers appear hypoechoic on ultrasound images, facilitating their identification preoperatively (15, 30).

Just as myocardial and skeletal muscle thicknesses are clinically relevant in cardiovascular and muscular conditions (33, 34), the cross-sectional area of the gastric antrum has been linked to various perioperative adverse events (25, 35). Consequently, preoperative measurements of TMP and CSA-ISMP of the gastric antrum were selected as primary indicators in our morphological analysis. We hypothesized that TMP and CSA-ISMP could serve as biomarkers for predicting PONV.

Our findings indicate that patients with thicker TMP were less likely to develop PONV during the 6~24 hours post-surgery period. Gastrointestinal muscle contraction is regulated by the autonomic nervous system and intrinsic enteric neurons (23, 36). Any disruptions affecting these systems may alter smooth muscle function over the long term (37–39). Therefore, the difference in TMP observed between patients with and without PONV may reflect variations in gastrointestinal tract functional status. Interestingly, we found no significant difference in TMP between patients with or without PONV within the first 6 hours after surgery, suggesting TMP may not significantly predict early PONV occurrence. This finding warrants validation in larger-scale studies.

Our findings indicate that patients with a larger CSA-ISMP are more susceptible to developing PONV within the first 6 hours post-operation compared to those with a smaller CSA-ISMP. This increase in CSA-ISMP not only reflects greater residual or gas presence in the gastric antrum but also suggests tissue thickening due to edema or chronic inflammation in the submucosal and mucosal layers prior to surgery (40, 41). However, there was no significant difference in CSA-ISMP between patients with and without PONV during the 6~24 hours post-operation period, suggesting CSA-ISMP lacks predictive value for PONV occurrence in this timeframe. Larger-scale studies are needed to validate these findings.

The proper functioning of the gastrointestinal tract is crucial for food grinding, mixing with gastrointestinal secretions, and propelling contents distally, essential for digestion and absorption (42). Alterations in the normal contraction rate of 3 peristaltic waves per minute—either slower (bradygastric movement) or faster (tachygastric movement), or a combination thereof—are associated with decreased gastric emptying or gastroparesis (43). Despite this, the relationship between FP and PONV remains unexplored. Our study revealed that patients in the F_0or>4_ group were more likely to experience PONV than those in the F_1~4_ group during the 6~24 hours post-surgery period, aligning with the concept that abnormal gastric motility patterns indicate increased susceptibility to PONV.

While excessive gastric residue or gas accumulation are generally indicators of heightened risk for reflux aspiration (44), their predictive value for PONV has not been previously investigated. Our study found no significant association between preoperative gastric residue or gas accumulation and the likelihood of developing PONV. Several factors may contribute to this result. Firstly, ultrasonography was performed in the supine position, potentially causing gastric content to spread widely along the gastric wall, making it challenging to distinguish from underlying gastric tissue. Secondly, the minimal presence of gastric content and gas in the preoperative fasting state increases difficulty in detecting subtle differences between patients. Lastly, the study’s limited sample size may have contributed to the low agreement rate between the two physicians. Future studies should aim to increase sample size and establish more stringent evaluation criteria to enhance accuracy and reliability in predicting PONV.

## 5 Limitations

There are several limitations in the current study. Firstly, although various established factors influencing PONV occurrence, such as gender, type of surgery, intraoperative inhalational anesthetics, and postoperative opioid use, were rigorously controlled, the generalizability of our findings to broader surgical populations requires further investigation. Secondly, this study did not delve into the underlying mechanism behind the observed phenomenon—specifically, why and how differences in preoperative gastric morphological characteristics correlate with varying incidences of PONV. Whether modifying preoperative gastric morphology could potentially reduce PONV incidence warrants further exploration. Additionally, this study did not assess the predictive value of gastrointestinal morphological parameters for the severity of postoperative nausea and vomiting. Lastly, being a single-center observational study with a limited sample size, future research should involve multicenter studies with larger cohorts to validate our results.

## 6 Conclusions

In conclusion, this study highlighted that patients with a larger CSA-ISMP were more susceptible to developing PONV within the initial 6 hours post-operation. Moreover, those with a thinner TMP and abnormal FP before surgery were at higher risk of experiencing PONV between 6 to 24 hours post-operation. These insights could enhance the precision of current PONV prediction methods and potentially lower the expenses associated with its prevention and management in future clinical practice.

## Data availability statement

The raw data supporting the conclusions of this article will be made available by the authors, without undue reservation.

## Ethics statement

The studies involving humans were approved by the Ethics Committee of Shanghai Fourth People’s Hospital Affiliated to Tongji University. The studies were conducted in accordance with the local legislation and institutional requirements. The participants provided their written informed consent to participate in this study.

## Author contributions

WQ: Conceptualization, Data curation, Funding acquisition, Investigation, Methodology, Visualization, Writing – original draft, Writing – review & editing. JY: Investigation, Methodology, Validation, Visualization, Writing – original draft. HL: Data curation, Visualization, Writing – original draft, Writing – review & editing. QS: Formal analysis, Investigation, Software, Validation, Writing – original draft. CL: Methodology, Validation, Writing – original draft. LZ: Formal analysis, Software, Validation, Writing – original draft. GB: Investigation, Software, Writing – original draft. GC: Conceptualization, Project administration, Supervision, Writing – review & editing. LX: Conceptualization, Data curation, Funding acquisition, Methodology, Supervision, Writing – review & editing.
