# Supplementary material for: Predictive value of preoperative ultrasonographic measurement of gastric morphology for the occurrence of postoperative nausea and vomiting among patients undergoing gynecological laparoscopic surgery
Source: Front Oncol. 2024 Jul 23;14:1296445. doi: 10.3389/fonc.2024.1296445 (PMC11300229; doi:10.3389/fonc.2024.1296445)
Supplement: Supplementary file 1 [file DataSheet_1.docx]

**Supplementary Table 1.** Description of interobserver variability and agreement

| CSA-ISMP_1_ (cm^2^) | CSA-ISMP_2_ (cm^2^) | *P_CSA_* | *R_CSA_* | *P_R-CSA_* |
| --- | --- | --- | --- | --- |
| 2.601±0.9556 | 2.525±0.9635 | 0.4984 | 0.7879 | <0.0001 |
| TMP_1_ (mm) | TMP_2_ (mm) | *P_TMP_* | *R_TMP_* | *P_R-TMP_* |
| 1.925±0.7873 | 1.826±0.675 | 0.2531 | 0.7946 | <0.0001 |

CSA-ISMP_1_, CSA-ISMP measured by Measurer 1

CSA-ISMP_2_, CSA-ISMP measured by Measurer 2

*P_CSA_*, P value of CSA-ISMP_1_ vs. CSA-ISMP_2_

*R_CSA_*, correlation coefficient between CSA-ISMP_1_ and CSA-ISMP_2_

*P_R-CSA_*, P value of correlation coefficient between CSA-ISMP_1_ and CSA-ISMP_2_

TMP_1_, TMP measured by Measurer 1

TMP_2_, TMP measured by Measurer 2

*P_TMP_*, P value of TMP_1_ vs. TMP_2_

*R_TMP_*, correlation coefficient between TMP_1_ and TMP_2_

*P_R-TMP_*, P value of correlation coefficient between TMP_1_ and TMP_2_

This data set demonstrates that there is no significant difference between measurements obtained by Measurer 1 and Measurer 2, and their data exhibit strong correlation.
